# Backtracking *NOM1::ETV6* fusion to neonatal pathogenesis of t(7;12) (q36;p13) infant AML

**DOI:** 10.1038/s41375-024-02293-9

**Published:** 2024-05-28

**Authors:** Pablo Bousquets-Muñoz, Oscar Molina, Ignacio Varela, Ángel Álvarez-Eguiluz, Javier Fernández-Mateos, Ana Gómez, Elena G. Sánchez, Milagros Balbín, David Ruano, Manuel Ramírez-Orellana, Xose S. Puente, Pablo Menéndez, Talia Velasco-Hernandez

**Affiliations:** 1 Departamento de Bioquímica y Biología Molecular, Instituto Universitario de Oncología (IUOPA), Universidad de Oviedo, Oviedo, Spain; 2 Centro de Investigación Biomédica en Red de Cáncer (CIBERONC), Madrid, Spain; 3 Josep Carreras Leukemia Research Institute, Barcelona, Spain; 4 Red Española de Terapias Avanzadas (TERAV), Instituto de Salud Carlos III, Madrid, Spain; 5 Instituto de Biomedicina y Biotecnología de Cantabria (IBBTEC), Universidad de Cantabria-CSIC, Santander, Spain; 6 Laboratorio de Oncología Molecular, Laboratorio de Medicina, Instituto Universitario de Oncología (IUOPA), Hospital Universitario Central de Asturias, Oviedo, Spain; 7 Department of Pediatric Hematology and Oncology, Hospital Infantil Universitario Niño Jesús, Autonomous University of Madrid, Madrid, Spain; 8 Unidad de Terapias Avanzadas, Laboratorio de Oncohematología, Fundación para la Investigación Biomédica del Hospital Infantil Universitario Niño Jesús, Madrid, Spain; 9 Instituto de Investigación Sanitaria La Princesa, Madrid, Spain; 10 Institució Catalana de Recerca i Estudis Avançats (ICREA), Barcelona, Spain; 11 Department of Biomedicine, School of Medicine, University of Barcelona, Barcelona, Spain

**Keywords:** Genetic translocation, Acute myeloid leukaemia, Oncogenesis

Acute leukemia is the most frequently diagnosed malignancy in childhood, with acute lymphoblastic leukemia (ALL) comprising approximately 80% of cases in children aged 0–18 years, and acute myeloid leukemia (AML) accounting for approximately 15–20% [1]. The early onset (0–10 years) of most childhood acute leukemias and the high concordance rate among monozygotic twins suggest a prenatal origin of the disease [1]. Indeed, the presence of preleukemic precursors in cord blood (CB) samples or Guthrie blood spots from children who later developed acute leukemia has been demonstrated in several studies [2, 3]. However, this has mainly been demonstrated experimentally for the most common cytogenetic subtypes, particularly in ALL.

The most prevalent genetic abnormality in infants (up to 1 year of age) with AML (iAML) is chromosome 11q23 translocations involving the *KMT2A* gene. This occurs at a higher incidence in children than in adults (38% *vs* 2%), with the highest incidence in infants (77%), suggesting that infant and adult AMLs are distinct biological entities. Notably, iAML may have an *in utero* origin [4], as demonstrated by long-established evidence of a prenatal origin for MLL rearrangements [5]. In fact, the t(8;21)(q22;q22) AML subgroup has also been associated with a prenatal origin [3]. However, studies of patients with AML in different molecular subgroups have failed to detect the corresponding genetic alteration in the respective CB or Guthrie blood spot samples [6], suggesting a less frequent prenatal origin of AML compared to ALL.

The t(7;12)(q36;p13) is a recurrent chromosomal rearrangement uniquely linked to AML. It ranks as the second most common abnormality in infants with AML, constituting nearly one-third of cases and associated with a dismal prognosis [7], with extremely poor survival rates and ineffective treatment by hematopoietic stem cell transplantation. Event-free survival rates (EFS) are 0–14% and overall survival (OS) 0–28%. However, more recent studies indicate more optimistic outcomes, with 3-year EFS rates of 43% and 3-year OS rates of 100%, albeit with high relapse frequencies [8]. The clinical manifestation of t(7;12) primarily presents as AML, although it has been diagnosed in a few cases as B-ALL or biphenotypic leukemia [8]. t(7;12)+ blasts often exhibit a poorly differentiated immunophenotype. Despite lacking a specific association with a French-American-British (FAB subtype), blasts are commonly classified as M0, M1, or M2 [8].

The breakpoint on chromosome 7 exhibits considerable heterogeneity, impacting the region 7q31-7q36, which is proximal to the motor neuron and pancreas homeobox 1 (*MNX1)* gene. There are cases reporting patients with canonical breakpoints and other harbouring non-classical translocations [8, 9]. This region is entirely translocated to the derivative chromosome 12 [7]. Simultaneously, the breakpoint on chromosome 12 is situated at position 12p13, disrupting the ETS variant transcription factor 6 (*ETV6*) gene in its 5’ region, specifically between exons 1 and 3 [8]. Notably, a significant portion of t(7;12) cases is reported in conjunction with specific aneusomies. The presence of one or more extra copies of chromosomes 8, 19, or 22 has been consistently observed [7], with trisomy 19 being particularly prevalent in over 70% of cases [8]. The concurrent occurrence of these numerical anomalies has been suggested as a potential factor in the development of leukemia. However, there is no clear evidence regarding the mechanistic advantage of acquiring these additional chromosomes, although some studies propose that it may lead to the overexpression of specific genes [8, 10]. In line with other cases of iAMLs, additional mutations are infrequent. A common feature of t(7;12) patients is *MNX1* overexpression, which has been studied extensively using in vitro and in vivo models attempting to recapitulate the biology of this disease [11]. *MNX1* overexpression does not lead to leukemic transformation of cord blood (CB) cells or adult mouse bone marrow (BM) cells, but impedes erythroid differentiation and encourages cellular senescence [12]. However, *MNX1* overexpression in fetal liver cells, but not in adult BM cells, leads to leukemic transformation in a retroviral mouse model [13]. *MNX1::ETV6*, independently of *MNX1* overexpression, does not confer self-renewal capacity or leukemogenic potential in a model involving transduced fetal liver cells. Only an in vitro myeloid biased was described in these assays [13]. Thus, the precise role of the *MNX1::ETV6* fusion transcript remains a subject of debate.

**Fig. 1:**
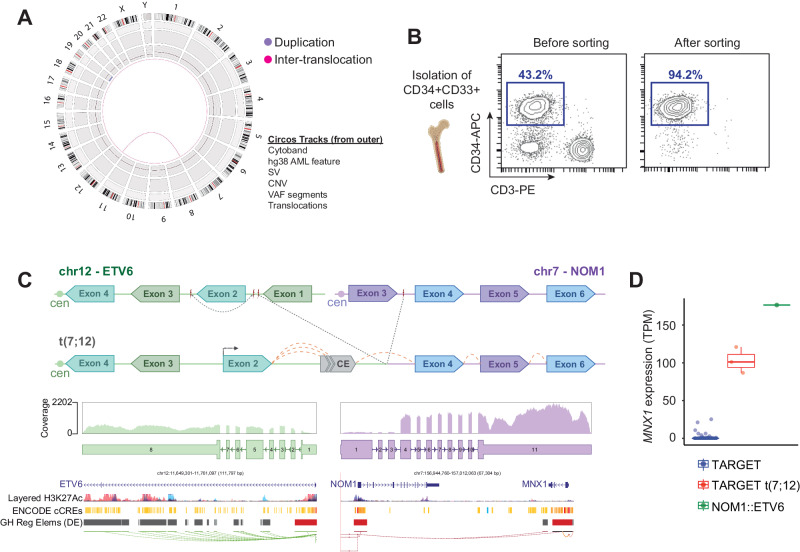
Characterization of a novel breakpoint in t(7;12) iAML. **A**. Optical Genome Mapping circos plot depicting the identified translocation in the diagnostic BM sample. Low confidence and non-somatic alterations are not displayed. Each circos track indicates (from outer ring): corresponding cytoband, hg38 AML feature, structural variants (SVs), copy number variants (CNVs), variant allele frequency (VAF) segments and translocations. **B**. Flow cytometry analysis of diagnosis BM sample before and after the AutoMACs separation of CD34 + CD33+ cells for DNA extraction. The purity of CD34 + CD33+ sorted cells was 94%. **C**. Genetic alterations identified by WGS and expression quantification of exons by RNA-seq. Top panels, representation of *ETV6* and *NOM1* exons surrounding the breakpoint in the reference genome and in the translocated allele (t(7;12)). Breakpoints for the t(7;12) as well as for the inversion affecting exon 2 of *ETV6* are indicated (cen, centromere). RNA-seq expression data showing the presence of a cryptic exon (CE) within intron 1 of *ETV6*. Dashed red lines show splicing events between exons in the t(7;12) allele. Middle panels, quantification of exon expression by RNA-seq showing increased expression of *NOM1* exons involved in the translocation. Bottom panels, location of annotated regulatory elements from UCSC browser near the involved *loci*. (cCREs, Candidate Cis-Regulatory elements; GH, Enhancers, and promoters from GeneHancer). **D**. Expression of *MNX1* gene in TARGET dataset samples and the analyzed BM diagnosis sample (*NOM1::ETV6*) (TPM transcripts per million).

Here we present a case of iAML characterized by the t(7;12) translocation, offering evidence supporting the neonatal origin of the disease. Additionally, trisomy 19 is identified as a secondary oncogenic event, likely occurring postnatally in the leukemogenesis process. A 5-months-old boy was diagnosed with AML, wherein 40% of the BM cells were immature blasts (MPO+, CD34+, CD38+, CD117+, CD13+, CD33+, CD123−/+, CD9−/+, HLADR+) displaying an aberrant expression of the non-myeloid markers CD7, CD2 and CD56. Cytogenetic analysis by optical genome mapping (OGM) revealed a karyotype 47, XY,t(7;12)(q36;p13),+19, with no additional structural or copy number variants (SVs and CNVs) (Fig. 1A). Recurrent mutations for *FLT3*, *NPM1*, and *TP53* genes were also ruled out. We performed WGS on AutoMACS-separated BM leukemic blasts (CD34 + CD33+) to characterize the breakpoint and genes involved in the translocation (Fig. 1B). The CD34-CD33- cell population was used as non-leukemic control cells. The analysis confirmed the presence of the translocation with breakpoints at positions chr7:156,958,910 (affecting intron 3 of *NOM1)*, and chr12:11,698,832 (within intron 2 of *ETV6)* (Fig. 1C). Additionally, we observed a 35 Kb inversion (chr12:11,719,998-11,754,857) affecting exon 2 of *ETV6* and located 20 Kb from the translocation breakpoint on chromosome 12. Reconstruction of the chimeric chromosome revealed that the fusion resulted in exons *3-*8 of *ETV6* in opposing transcriptional orientation in relation to exons 4-11 of *NOM1*, except for exon 2 of *ETV6*, which showed the same transcriptional orientation as *NOM1* due to an inversion (Fig. 1C). Further analysis revealed a very low mutational burden, with 19 substitutions and indels, none of them affecting coding regions (data not shown), and confirmed the presence of trisomy 19, and a subclonal deletion of the short arm of chromosome 21.

In parallel, we performed RNA-seq of the BM blast cells to explore the functional consequences of the t(7;12) translocation. Analysis confirmed the existence of a chimeric gene connecting exon 2 of *ETV6* to a cryptic exon located within intron 1 of *ETV6* and containing multiple splicing acceptor sites, and the 3’-end of this cryptic exon was joined to exon 4 of *NOM1* (Fig. 1C). Consistently, expression analysis revealed pronounced overexpression of the exons of *NOM1* in the chimeric transcript compared to exons 1-3. By phasing single-nucleotide polymorphisms (SNPs) surrounding the breakpoint and SNPs within the exons, we identified strong allele-specific expression. The variant allele frequency (VAF) for the SNP rs11765440, located within the 3’UTR of *NOM1* and in phase with the translocation, was 95% by RNA-seq, whereas WGS VAF was only 50% (data not shown). This suggests the preferential overexpression of the chimeric allele in these cells. In contrast, a SNP in exon 3 of *ETV6* phased with the breakpoint showed reduced expression of this *ETV6* allele (RNA-seq VAF: 10%, WGS VAF: 57%), supporting the notion that the translocation results in *ETV6* silencing.

Despite the observed overexpression of *NOM1*, the mechanism by which this transcript might contribute to leukemogenesis remains unclear, particularly as the cryptic exon within intron 1 of *ETV6* had many splice acceptor sites, resulting in the absence of an open reading frame for the chimeric transcript. Therefore, considering the physical proximity of *NOM1* to *MNX1* ( < 32 Kb), a pivotal player in the canonical t(7;12) translocation, we studied whether the translocation in *NOM1* influenced *MNX1* expression. RNA-seq analysis showed that *MNX1* was highly expressed in this sample (176 transcripts per million) and was among the top 2% highly expressed genes. To compare its expression with other AML samples with or without the t(7;12) translocation, we integrated our expression dataset with that of the TARGET [14]. This analysis revealed that the sample from our patient had the highest expression of *MNX1* relative to the dataset, followed by three additional cases from TARGET that were positive for t(7;12) (Fig. 1D). These results strongly suggest that, despite involving *NOM1*, t(7;12) has a major effect on *MNX1* expression, as it was previously described [8]. Indeed, the translocated intron 1 of *ETV6* contains numerous enhancers and regulatory elements that may drive *MNX1* overexpression, mimicking the molecular mechanism described for canonical t(7;12) cases [11]. Of note, a recent study points to an enhancer-hijacking event activating the *MNX1* promoter from the *ETV6* locus as an explanation for the *MNX1* overexpression in this AML subtype [15]. Overall, these observations support that *MNX1*, rather than *NOM1*, is most likely the driver event of the disease, akin to other t(7;12) leukemias [13].

**Fig. 2:**
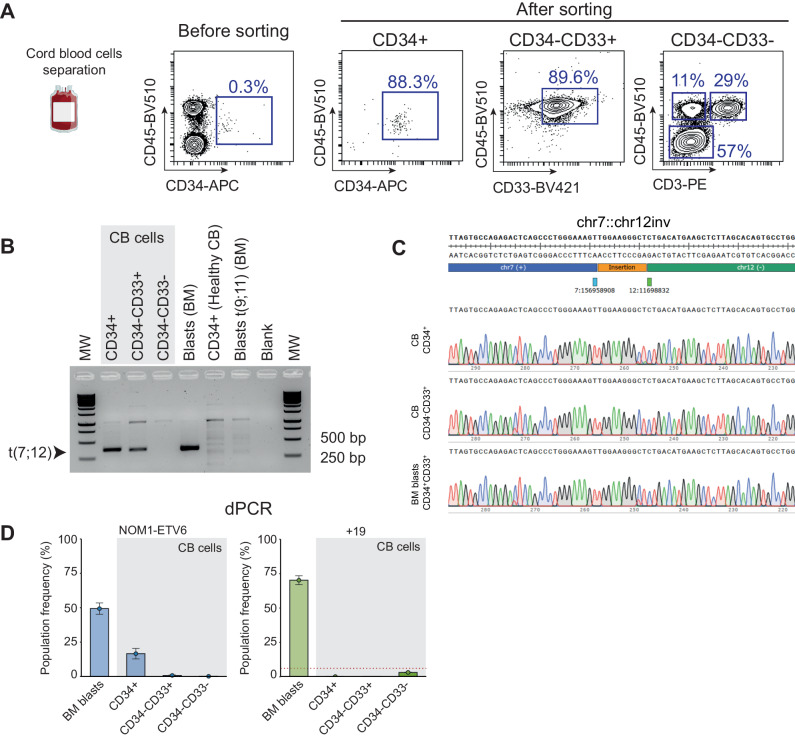
Neonatal origin of the t(7;12) iAML. **A** Flow cytometry analysis of CB cells before and after AutoMACs separation to isolate the indicated hematopoietic populations for DNA extraction. FACS purity of separated CD34+ HSPCs and CD34-CD33+ myeloid cells was ~90%. Non-myeloid cells were mainly comprised by erythroblasts (57%), T cells (29%), and non-T cell leukocytes (11%). **B** PCR amplification of the t(7;12) breakpoint in the different hematopoietic populations separated from CB and in BM blasts of the patient. Unrelated CB cells and a t(9;11) AML cells were used as controls. **C** Sanger sequencing of the amplified t(7;12) PCR band from the indicated populations. **D** dPCR analysis of the *NOM1::ETV6* fusion and trisomy 19 in the different indicated populations. Negative controls were included to identify the threshold (red line) above which a sample could be considered positive for +19. CB cord blood; BM bone marrow; MW molecular weight.

To investigate the potential neonatal origin of the translocation, we examined the patient’s cryopreserved CB cells collected at birth. CB cells separated and enriched for hematopoietic stem and progenitor cells (HSPCs) (CD34+), myeloid progeny (CD34-CD33+) and more differentiated non-myeloid cells (CD34-CD33-) (Fig. 2A). PCR amplification of the t(7;12) was observed in both CD34+ HSPCs and the myeloid progeny, but not in differentiated non-myeloid cells (Fig. 2B). The identity of the translocation product was confirmed by Sanger sequencing and was identical to that detected by WGS in the diagnosis BM sample (Fig. 2C). We then performed microfluidic digital PCR (dPCR) on DNA from these populations to quantify the proportion of cells with the translocation (Fig. 2D). The t(7;12) translocation could be detected in 18% of CD34+ cells, whereas <1% of CD34-CD33+ and CD34-CD33- cells were positive for the translocation. Similarly, dPCR was used to evaluate the copy number status of chromosome 19, the other major event identified by OGM and WGS and often associated to t(7;12) iAML. Notably, trisomy 19 was below the detection limit in all CB populations but was readily detected in the diagnosis BM sample, indicating that chromosome 19 duplication is a secondary event that occurred postnatally. Altogether, our results provide the first evidence for the neonatal origin and the cell compartment-of-origin of the translocation t(7;12). Ultimately, functional gain-of-function and or gene-editing experiments with t(7;12)/*ETV6::NOM1* fusion in CD34 + HSPC cell fractions may provide fundamental knowledge about the leukemogenic potential and precise the cell-of-origin of t(7;12) iAML.

### Supplementary information

Supplemental Information.
