# Supplementary material for: Backtracking NOM1::ETV6 fusion to neonatal pathogenesis of t(7;12) (q36;p13) infant AML
Source: Leukemia. 2024 May 28;38(8):1808–12. doi: 10.1038/s41375-024-02293-9 (PMC11286527; doi:10.1038/s41375-024-02293-9)
Supplement: Supplementary file 1 — Supplemental Information. [file 41375_2024_2293_MOESM1_ESM.pdf]

Supplemental Information

Backtracking *NOM1::ETV6* fusion to neonatal pathogenesis of t(7;12) (q36;p13) infant AML

Pablo Bousquets-Muñoz*, Oscar Molina*, Ignacio Varela, Ángel Álvarez-Eguiluz, Javier Fernández-Mateos, Ana Gómez, Elena García-Sánchez, Milagros Balbín, David Ruano, Manuel Ramírez-Orellana, Xose S. Puente[#], Pablo Menéndez[#] and Talia Velasco-Hernandez[#].

Supplemental Material and methods

Patient sample

The sample was collected during routine diagnostic procedures after written informed consent from the parents. The study was approved by the Institutional Review Board of Hospital Clinic Barcelona (HCB/2018/0020). AML mononuclear cells were cryopreserved in fetal bovine serum (FBS) with 10% dimethyl sulfoxide (DMSO) in liquid nitrogen. CB cells were frozen in culture medium with 10% DMSO and maintained at the Bebé Vida biobank (Portugal) from birth and recovered under parents' request.

Optical genome mapping

Ultra-high molecular weight DNA was extracted from AML cells obtained from bone marrow (BM) at diagnosis using the SP-G2 Bone Marrow Aspirate DNA Isolation Kit and labelled with DLE-1 enzyme according to Bionano Prep Direct Label and Stain-G2 (DLS-G2) (Bionano Genomics Inc.). DNA quantification was performed using a Qubit dsDNA Assay BR or HS Kit (ThermoFisher Scientific, #Q32853; Q32854) with small modifications by a Quibit 4.0 Fluorometer (Invitrogen). The DNA was loaded into the Saphyr Chip G3.3 (#20440) and 1,500 Gbp was scanned on the Saphyr platform (Bionano Genomics Inc.). The Rare Variant Pipeline (RVP) and Variant Calling were performed using Bionano Solve software (v3.7). Visualization and reporting of structural variants and CNV were performed using the Bionano Access (v1.7), using GRCh38 as reference genome, with the following filters: 1) Structural variant (SV) filters: overlap precision 3 kpb, non-masked SV only, variant allele frequency (VAF) between 0-1, SV found in at least 5 molecules, 0% of SV found in control database, SV found in self-molecules, recommended confidence scores and minimum size of 100,000 pb for insertions, deletions, inversions and duplications. 2) CNV filters: overlap precision 15 kpb, all variants found, recommended confidence scores, minimum size 500,000 bp and non-masked CNV only. 3)

Aneuploidy filters: all and recommended confidence scores. A specific browser extensible data (BED) file was used to visualize genetic events related to pediatric AML.

Cell enrichment by AutoMACS and flow cytometry

Frozen BM and cord blood (CB) cells were thawed and hematopoietic and stem progenitor cells (HSPCs) were isolated using magnetic beads conjugated with anti-human CD34 (130-046-703) and/or anti-human CD33 (130-045-501) using the AutoMACS Pro Separator (Miltenyi Biotec) according to manufacturer´s instructions. Purity of isolated populations was confirmed by flow cytometry stained with the following antibodies for 30 min at 4ºC: hCD34-APC (581), hCD33-BV421 (WM53), hCD3-PE (UCHT1), hCD19-FITC (HIB19), hCD45-BV510 (HI30) (all from BD Biosciences). Cells were washed with PBS + 2% fetal bovine serum (FBS) after staining and analysed using a FACSCanto™-II flow cytometer equipped with FACSDiva™ software.

Whole-genome sequencing analysis

DNA was extracted from cells after AutoMACs enrichment using the DNeasy Blood & Tissue Kit (Qiagen) and 1 µg of genomic DNA for each cell population was used for whole genome sequencing (WGS) (Novogene). Raw reads were aligned with decoy and virus sequences (GRCh38.d1.vd1) using bwa-mem2[16], and somatic variants and indels were identified with RFcaller[17], CNVs using Battenberg[18] and structural variants with GRIDSS2[19].

RNA-sequencing

RNA was extracted from AML BM cells after AutoMACs enrichment using a Maxwell RSC simply RNA Cells Kit (Promega) and used for RNA sequencing (Novogene). Fusion gene calling was carried out with Arriba (v2.4.0)[20]. Additionally, raw reads were aligned with STAR[21] following GDC DR15plus RNA-seq alignment pipeline, to facilitate harmonization with

TARGET's datasets[22]. GENCODE v22 was used to annotate the transcripts, and gdc_rnaseq_tools augment_star_counts was used for count normalization (NCI-GDC/gdc-rnaseq-tool). The TARGET-AML transcriptomic dataset was retrieved from GDC data portal (portal.gdc.cancer.gov).

Allele-specific expression affecting *NOM1* and *ETV6* aberrant isoforms was assessed by phasing single nucleotide polymorphisms (SNPs) around the breakpoint and SNPs located at the exons, with IMPUTE2[23]. These SNPs were used as proxies of wild-type and translocation-derived isoform expression.

Validation of t(7;12) by polymerase chain reaction

To detect the *NOM1::ETV6* fusion-gene in the different isolated cell populations from the CB sample, a polymerase chain reaction (PCR) was performed using primers to detect both t(7;12) breakpoints: Chr7-Chr12 inv: Chr7-Forward GCCCAGTGCCCCTCAAACCA; Chr12-inv-Reverse GGCCTCCCAACCTAGACACCC. The identity of the PCR products was confirmed by Sanger sequencing.

Microfluidic digital PCR

Microfluidic digital PCR (dPCR) was used to assess absolute amounts of the reciprocal t(7;12) and trisomy +19. Assays were performed on leukemic blasts purified from BM as well as CD34+ HSPCs, CD33+CD34- myeloid cells and CD34-CD33- non-myeloid cells derived from CB. Custom multiplex TaqMan MGB probe-based assays were designed using Primer Express™ Software v2.0.0 (see table below) spanning the junction of t(7;12) and within *SIPA1L3* for trisomy +19. *SF3B1* was used as endogenous control.

Target	Type	Sequence
t(7;12)	Fwd	5'-GCAGTGTCTGGTTTACCTGCTG-3'
t(7;12)	Rev	5'-CAGGCACTGTGCTAAGAGCTTC-3'
t(7;12)	Probe	5'-FAM-TCAGAGCCCTTCCAA-3'-MGB
SIPA1L3 (tri19)	Fwd	5'-AGGGAATGTTGGCAAAACAGC-3'
SIPA1L3 (tri19)	Rev	5'-GCCAGCTCCTAACAGCGACTT-3'

SIPA1L3 (tri19)	Probe	5'-Cy5-ACGACCAGCCATCT-3'-MGB
SF3B1 (endogenous control)	Fwd	5'-CCTACGTTTGCTTGGCCTCT-3'
SF3B1 (endogenous control)	Rev	5'-GAGCCTCTATTTGGTCCCAATG-3'
SF3B1 (endogenous control)	Probe	5'-VIC-GGCATTGTGGAGCAA-3'-MGB

Reaction mixtures contained 16 µM of each primer, 8 µM of the target probe and 4 µM of the control probe, as well as 20 ng of genomic DNA. They were assembled into standard 96-well PCR plates as per manufacturer's instructions, and samples were analysed in two independent assays (FAM-NOM1::ETV6 + VIC-SF3B1; Cy5-SIPA1L3 + VIC-SF3B1), in duplicate, along with wild-type genomic DNA and no-template control (NTC). The manufacturer-specified workflow was followed for the reaction and image acquisition.

The dPCR run comprised an initial heat activation at 95ºC for 2 minutes. Then, a 2-step cycling (40 cycles: 95ºC, 15s; 58ºC, 1 min) was applied for detection of the chimeric *NOM1::ETV6* gene, while for copy number a 3-step cycling protocol was used (40 cycles: 95ºC, 15 s; 58ºC, 45 s; 72ºC, 45 s). Partitions were imaged with 500 ms exposure time for green (FAM™) and yellow (VIC®) target channels, or 600 ms for the crimson channel (Cy5 fluorophore), and gain set to 6 or 8, respectively. At least 25,400 valid, analysable reactions were performed for each sample. Data were analysed by using the QIAcuity® Software Suite 2.2.0.26 (Qiagen) according to Poisson statistics based upon the total number of positive partitions, and absolute quantifications of copies/microliter of each target present in samples were registered. The determination of CNVs also consisted of quantification of changes in copy number (CN)/genome of each target assay in relation to a defined reference sample and/or reference assay.

Data access

Newly generated whole-genome sequencing (WGS) and RNA-sequencing (RNA-seq) data have been deposited at EGA with accession number EGA50000000179.

Supplemental References

16. Vasimuddin M, Misra S, Li H, Aluru S. Efficient Architecture-Aware Acceleration of BWA-MEM for Multicore Systems. 2019 IEEE International Parallel and Distributed Processing Symposium (IPDPS); 2019:314-324.

17. Diaz-Navarro A, Bousquets-Munoz P, Nadeu F, et al. RFcaller: a machine learning approach combined with read-level features to detect somatic mutations. *NAR Genom Bioinform*. 2023;5(2):lqad056.

18. Nik-Zainal S, Van Loo P, Wedge DC, et al. The life history of 21 breast cancers. *Cell*. 2012;149(5):994-1007.

19. Cameron DL, Baber J, Shale C, et al. GRIDSS2: comprehensive characterisation of somatic structural variation using single breakend variants and structural variant phasing. *Genome Biol*. 2021;22(1):202.

20. Uhrig S, Ellermann J, Walther T, et al. Accurate and efficient detection of gene fusions from RNA sequencing data. *Genome Res*. 2021;31(3):448-460.

21. Dobin A, Davis CA, Schlesinger F, et al. STAR: ultrafast universal RNA-seq aligner. *Bioinformatics*. 2013;29(1):15-21.

22. Ma X, Liu Y, Liu Y, et al. Pan-cancer genome and transcriptome analyses of 1,699 paediatric leukaemias and solid tumours. *Nature*. 2018;555(7696):371-376.

23. Howie B, Marchini J, Stephens M. Genotype imputation with thousands of genomes. *G3 (Bethesda)*. 2011;1(6):457-470.
